# Application of an Artificial Neural Network in the Modelling of Heat Curing Effects on the Strength of Adhesive Joints at Elevated Temperature with Imprecise Adhesive Mix Ratios

**DOI:** 10.3390/ma15030721

**Published:** 2022-01-18

**Authors:** Jakub Szabelski, Robert Karpiński, Anna Machrowska

**Affiliations:** 1 Department of Computerization and Production Robotization, Faculty of Mechanical Engineering, Lublin University of Technology, Nadbystrzycka 36, 20-618 Lublin, Poland; 2 Department of Machine Design and Mechatronics, Faculty of Mechanical Engineering, Lublin University of Technology, Nadbystrzycka 36, 20-618 Lublin, Poland; a.machrowska@pollub.pl

**Keywords:** artificial neural networks, LSTM, modelling, adhesive joints, epoxy, resin, hardener, mix ratio, temperature degradation

## Abstract

This paper is a discussion of the results of tests intended to (i) estimate the effects of component mix ratios and heat curing of an adhesive joint on the tensile strength, and (ii) to determine the adhesive component mix ratio for which heat curing is insignificant to the strength of adhesive butt joints. Experimental tests were carried out at ambient temperature and elevated temperature during which adhesive butt joints were loaded with a tensile force until failure. The variables were the mix ratio of epoxy adhesive components and the application of heat holding at the adhesive curing stage. An LSTM (long short-time memory) forecast was used to determine the point corresponding to the mix ratio of adhesive components at which heat holding of the adhesive joint no longer has a positive and significant importance to the final tensile strength of the joint.

## 1. Introduction

Joining with adhesives which are sometimes underestimated is a major complement to the traditional techniques of joining materials. Adhesive joining or bonding is applied not only where the operating conditions of a joint do not require it to have high strength but also for the joining of structural materials, such as metals, and beyond simple applications, which means the aerospace industry, for example, to produce structures which require superior strength to mass ratios [1,2].

There is a number of requirements to be met for the final adhesive joint to be produced properly, meaning strong and durable. The requirements include proper pretreatment of the surfaces to be joined, preparation of the adhesive, and suitable conditions for adhesive curing. The final quality of an adhesive joint also depends on its operating conditions, both in terms of the behaviour of the joint, the joint’s operating temperature and its range of variation, the operating humidity, and more. These issues belong to different scientific disciplines, thus adhesive bonding can be called an interdisciplinary process, which combines chemistry, physics, and mechanics [3,4,5,6,7,8,9].

The sheer diversity of adhesive compositions in use requires engineers to understand the methods and parameters of adhesive curing. Some adhesives become cured by chemical reactions while other adhesives develop their properties by evaporation of solvents and for some adhesives, curing is achieved by solidification of their melts. In this selection of the basic groups of adhesives, subgroups of adhesives can be refined by the detailed mechanism of curing. Chemical reaction-cured adhesives include two-component adhesives, single-component adhesives activated with catalysts or hardeners, moisture-cured adhesives, UV-cured adhesives, and substrate-catalysed anaerobic adhesives. Between the adhesive groups, the adhesive joints produced with them vary in performance, but first they vary in the recommendations and processes for proper fabrication of adhesive joints [7,10,11].

A study of chemically cured two-component adhesives, namely epoxy materials, revealed that the quantitative mix ratio of the epoxy resin to its hardener is significant to the final performance characteristics of the adhesive. If there is too little of a hardener per unit of resin, incomplete polymerisation may occur, whereas too much of the hardener may result in brittleness and fragility of the cured compound, leading to corrosion of the metallic interfaces [12,13,14]. Other characteristics of adhesives, such as resistance to heat or rigidity, may also change with deviations from the specified resin-to-hardener ratios or when admixtures are used [15,16,17,18,19,20]. This is due to the different degree of crosslinking, i.e., the case of an incomplete or different mode of curing the polymeric adhesive material when there is a shortage of hardener; on the other hand, when there is an excess, a certain amount of unreacted hardener remains in the adhesive.

One of the ways for optimising the results of adhesive joining is curing of the adhesive while holding the joint at heat [4]. The heat holding is intended to change the physical properties of the adhesive joint by altering the crosslinking performance. The technical data sheets of adhesives are usually rather vague in specifying that as the curing temperature increases, so does the joint strength, while the curing time is reduced [21,22,23]. 

An analysis of mechanical performance durability is critical to the estimation of adhesive joint life and the design engineering of modern materials. This is especially important because these materials are often required to withstand elevated temperature conditions and the adhesive strength of epoxy is decreases with increased temperature [24]. However, a large number of experimental tests is often infeasible, for example, because of the constraints of time and costs. One of the solutions can be the application of computer methods, such as the finite element method (FEM) [25,26,27,28,29,30], the boundary element method (BEM) [31,32,33,34], predictive modelling [35,36,37,38,39], and data analytics [40,41,42,43,44,45]. Mathematical modelling with a modest dataset acquired may help to determine the relationships between the individual parameters and mechanical properties, to identify the most promising direction of research, to reduce the number of physical tests, and to markedly reduce the time and costs of research.

Another way of approaching the problem of eliminating quality level deviations in manufacturing processes from the desired values may be the Taguchi method, a technique of optimising process parameters by reducing the variation in the process and examining how different parameters can affect both the mean and variance of the outcome characteristics of the process, as well as which variables have a significant effect. The method defines product quality as the difference between the characteristics of the process result and the value to be achieved (target), which is defined as a function. The Taguchi method uses a procedure that applies orthogonal arrays to statistical design experiments to obtain good results with a minimum number of experiments. This can reduce the cost and time required for the experiment. The objective function of this experimental matrix is the signal-to-noise (S/N ratio. It is used to measure the yield characteristics of the process and the percentage contribution of the process parameters through an analysis of variance. If these characteristics are continuous, then the S/N ratio can be classified into three categories: nominal-the-best, smaller-the-better, and larger-the-better characteristics. The optimal level of process parameters for this optimisation is the level that produces the largest S/N ratio transformation [46]. There are known studies in the field of polymer engineering in which this method was applied [47,48], however, the subject of this paper has not been studied with this method so far.

Response Surface Methodology (RSM) is a set of mathematical and statistical methods for experimental model building and the optimisation of any process in which the response of interest is influenced by several independent factors and their interactions. Central composite design (CCD) is the most suitable experimental design used in RSM, which helps to optimise effective parameters with a minimum number of experiments, as well as to study the interaction between parameters. As it allows for evaluation of the influence of multiple factors and their interactions on one or more response variables, it is a very useful method [49,50,51].

This paper was an attempt at analysing the accuracy of the results produced with the tools for modelling the effects of deviation from the specified adhesive mix ratios on the potential reduction of the performance of an adhesive in operation at temperatures higher than ambient. The modelling tool considered was artificial neural networks (ANNs) capable of memorising and processing information of random input data sequences. The tasks of classification and forecasting was based on a full dataset used as the input data for teaching the ANN. ANNs are designed to process data by classifying it into specific sequences. When solving problems with artificial neural networks, the type and operating principle of an ANN must always be considered or the ANN can be ‘overtrained’ [52,53]. Overtraining is symptomatic by overt matching of the ANN to the teaching data. Specific sequences and structures present in one-off runs can also be memorised by ANNs. Although the capacity of ANNs for information acquisition is very useful, certain problems benefit just as well from the capacity of an ANN to ‘forget’ past states. An example of this application is an information stream which contains a sequence within which subsequences exist with a variable structure. Given these types of processing tasks, a need emerged to build ANNs capable of resetting the memorised states by setting their values to zero without any need for a system operator to intervene and manually delete the states. This is what led to artificial recurrent neural networks (RNN) with the architecture termed LSTM (long short-time memory) [54]. 

**Figure 1:**
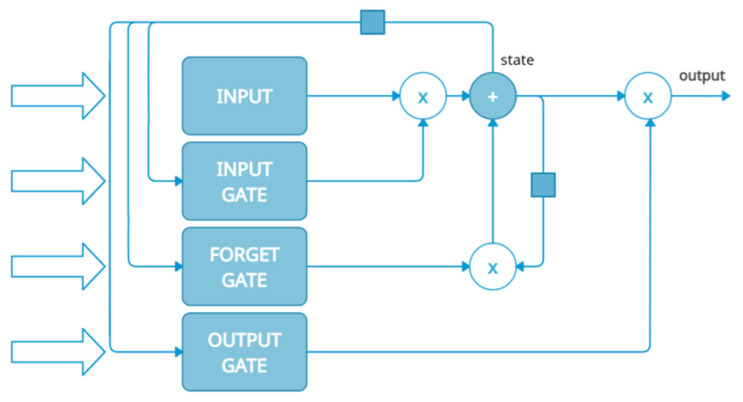
LSTM information processing diagram.

The essence of LSTM is feedback loops existing between the basic network units, which are the memory cells. Another distinguishing feature of LSTM is that it has specific logic gates: an input gate, a forget gate, and an output gate. The input data is calculated by regular artificial neuron units. The accumulation of their values to a state is governed by an input gate, which learns to protect the error flow present in the memory cells against interference from irrelevant input data [55]. The steady, non-decaying, and non-increasing error flow is managed by a feedback loop and the forget gate, which controls the maintenance of the stability of the weight. The output gate opens and closes access to the steady error flow, learning to protect other memory cells against interference from their currently irrelevant contents. A diagram of information processing in LSTM [56] is shown in Figure 1.

An LSTM RNN was applied in this work to complete the reliable forecast of the full-strength characteristics and maximum breaking forces of specimens. An information sequence containing several sets of full-strength characteristics was a dataset for which the capability to control the changes in the data structures of a narrower range was relevant. 

In summary, the study analysed the results of tensile strength tests of adhesive joints made using an adhesive with a deviation from the manufacturer’s recommended A/B ratio (resin/hardener). Two methods of joint curing were tested: at ambient temperature and with additional heating. Samples were tested at ambient and elevated temperatures. LSTM RNN analyses were performed to determine the limiting proportion of adhesive components for which the reheating process at the stage of the adhesive crosslinking of polymeric material would not influence the improvement of the mechanical parameters of the adhesive bond.

## 2. Materials and Methods

### 2.1. Materials and Sample Preparation

**Figure 2:**
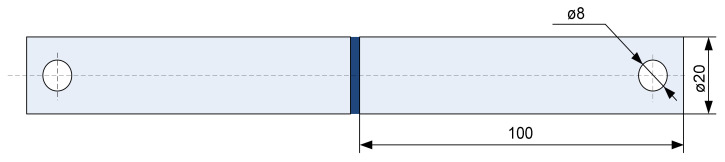
Cylindrical specimen comprising two bars butt-joined by adhesive bonding.

Experimental tests were performed on adhesive butt joints made with ø20 × 100 mm cylindrical specimens made of grade 1.0037 steel (EN 10025:2019–S235JR; Figure 2). The butt surfaces were pretreated per EN 13887:2005 (Structural adhesives—Guidelines for surface preparation of metals and plastics prior to adhesive bonding) with steady processing parameters to ensure the geometric repeatability of the surface pretreatment for all specimens.

**Figure 3:**
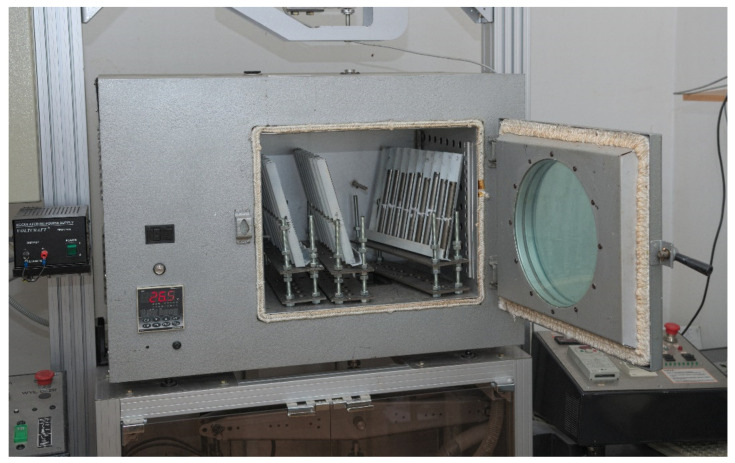
Self-alignment fixture in heating chamber.

The specimens were bonded with Loctite Hysol 9492 (Henkel, Düsseldorf, Germany), a commercially available, two-component adhesive composition. The adhesive was supplied in a manually emptied cartridge which provides a constant resin-to-hardener mix ratio of 2:1 [21]. To precisely estimate the weight ratio, the components were mixed manually after precise dispensing of the correct amounts by weight. The adhesive was mixed by hand and degassed in a vacuum chamber. The surfaces of the steel specimens to be adhesively bonded specimens were degreased with Loctite 7036 [57]. The specimens were fabricated by adhesive joining under an identical pressure (approx. 8 Pa) to obtain identical joints of a constant adhesive thickness of 0.08 mm in all specimens. The adhesive joints were cured according to the test plan (Table 1) with one of the two methods: a long cure at ambient temperature (3 days/25 °C) and a fast cure with heat holding (1 h/100 °C). To ensure the axial alignment of the upper and bottom specimen halves, a self-alignment fixture was used (a stand with vee blocks—Figure 3). Nine specimens were fabricated for each tested combination of the mix ratio, curing method, and planned strength testing temperature. The flashes of the adhesive were not removed, as permitted by EN 15870:2009 [58]. In the presented study, the degree of curing of the adhesive was not analysed for the conditions considered, assuming that the resulting inaccuracy of the proportions of the components of the adhesive is an accidental, unintentional error occurring at the stage of preparation of the adhesive and resulting in an unintentional change in the final strength properties of the joint made with such an adhesive. Crosslinking degree is of course an important factor in a detailed explanation of the mechanism of changes in joint strength.

### 2.2. Mechanical Testing 

**Figure 4:**
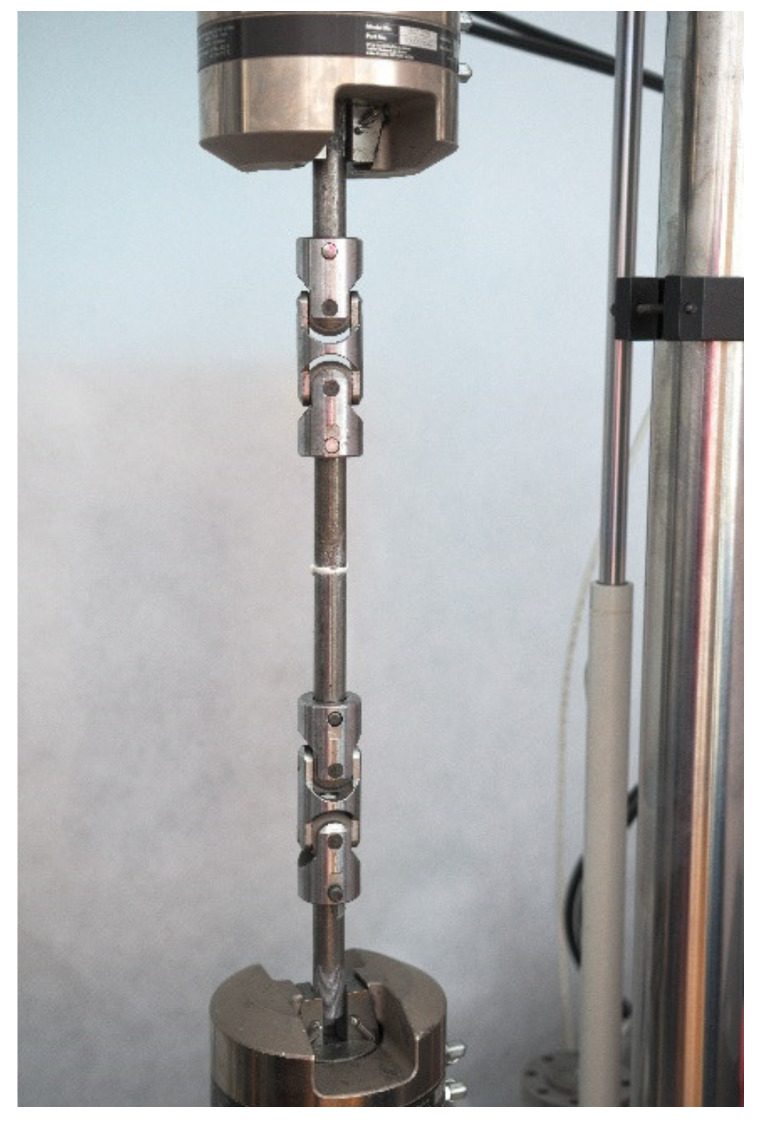
Fixtures with clamps (double-Cardan universal joint).

The produced specimen joints were tensile-tested. This was done in fixtures with clamps on both ends of the specimen to ensure that no bending moment was introduced during the tensioning (Figure 4). The test plan provided for different operating conditions of the adhesive joints, thus the specimens were tested in ambient temperature and elevated temperature (70 °C). The specimens tested at the elevated temperature were heated in an oven so that their entire volume was heated to the planned temperature level. Each specimen was tensioned at a steady rate of 4 mm/min and the failure force of the adhesive joint was recorded and converted to the joint strength by dividing the failure force by the cross-sectional area of the adhesive joint. For each of the tested adhesive joints, its failure mode was recorded with a total of three failure mode types: cohesive failure (within the adhesive joint), adhesive failure (between the adhesive layer and the bonded material), and mixed failure according to ISO 10365 Standard—Designation of main failure patterns [59] (Table 2).

### 2.3. Statistical Analysis

To verify if there were significant differences in the strength values between the heat-held and the non-heat-held adhesive joints, the experimental test results were analysed statistically [4,60] in the Statistica 13 suite from TIBCO Software Inc. (2017; Palo Alto, CA, USA). The significance level assumed was α = 0.05. A selection of strength test results (in pairs) was analysed using:

- The Shapiro–Wilk W-test (for the normality of distribution in the produced series of resultant joint strength test values);
- The tests by Fisher, Levene, and Brown–Forsythe (for the equality of group variances); and
- Student’s t-test (for the results characterised by normality of distribution and equality of variances to analyse the equality of the average resultant joint strength values at the adopted significance level).

### 2.4. Long Short-Term Memory Artificial Recurrent Neural Network

**Figure 5:**
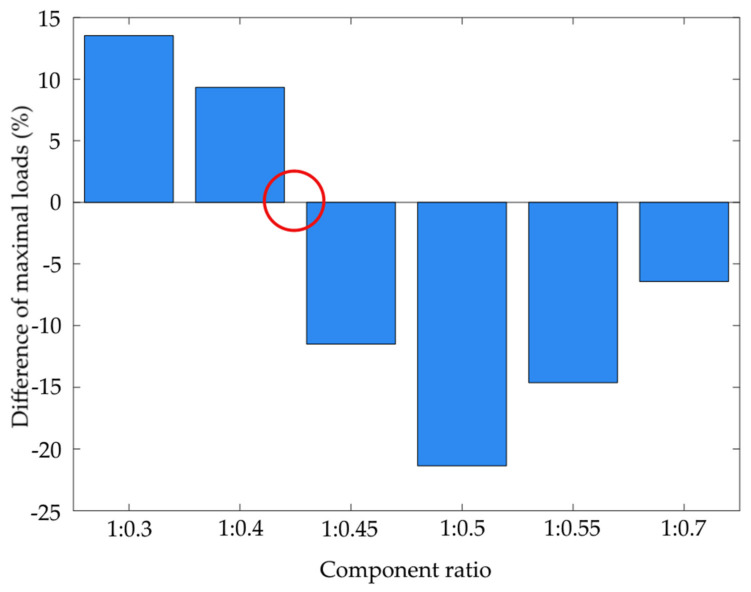
Relative change of the average static strength between the heat-held cured joints and the ambient temperature-cured joints.

The recorded experimental test data was analysed with two approaches: first, a set of averaged data was tested, and second, the processing was run on the samples at random within the assumed range of adhesive composition mix ratios. The common core of both approaches is the generic diagram of signal processing, consisting of the determination of the zero crossings for the differential maximum breaking force vs. the selection of component mix ratios (Figure 5). The columns show the values of the differences of the maximum destructive loads between the heated and non-heated joints, expressed as a percentage increase/decrease in relation to the non-heated joint load. The red circle marks the assumed area of the sought component ratio. Generally speaking, the test data, which included a specific mix ratio of the applied adhesive components, was analysed by forecasting the full-strength characteristics. The input data was the strength characteristics for the mix ratios at which the static strength was found to be reduced after heat-held curing. The sequence of input data was set from the highest to the lowest hardener ratio: 1:0.7; 1:0.55; 1:0.5; 1:0.45; 1:0.4; and 1:0.3.

**Figure 6:**
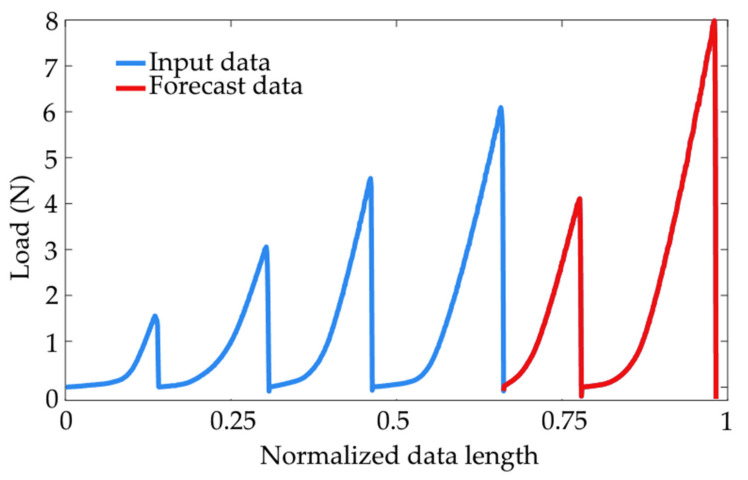
Range of forecast-processed data.

The signal processing diagram for the first approach involved preprocessing by resampling and averaging the data. The trends within the specified mix ratios were forecast. The declared teaching data was the averaged trends with the hardener ratio order from the highest to the lowest. Here, the ratios were 1:0.7; 1:0.55; 1:0.5; and 1:0.45, equal to 67% of the entire signal length (Figure 6). The remainder of the signal which included two full-strength characteristics (for mix ratios 1:0.4 and 1:03) were forecast with a DLN LSTM algorithm (Deep Learning Networks—Long Short-Term Memory). For the downstream data processing, the experimental test data was also included for mix ratios 1:0.4 and 1:0.3 (independent of the forecast). Based on the produced test results, the maximum breaking forces, namely *Lmax*, were determined for the heat-held cured joints (*LmaxH*) and the ambient temperature-cured joints (*LmaxN*). Based on the breaking force determined for the values obtained by experimental testing (*LmaxT*) and the values from the forecast characteristics (*LmaxF*), the following differences were determined:

(1) $$L_{maxTH}-L_{maxTN}$$

(2) $$L_{maxFH}-L_{maxFN}$$

**Figure 7:**
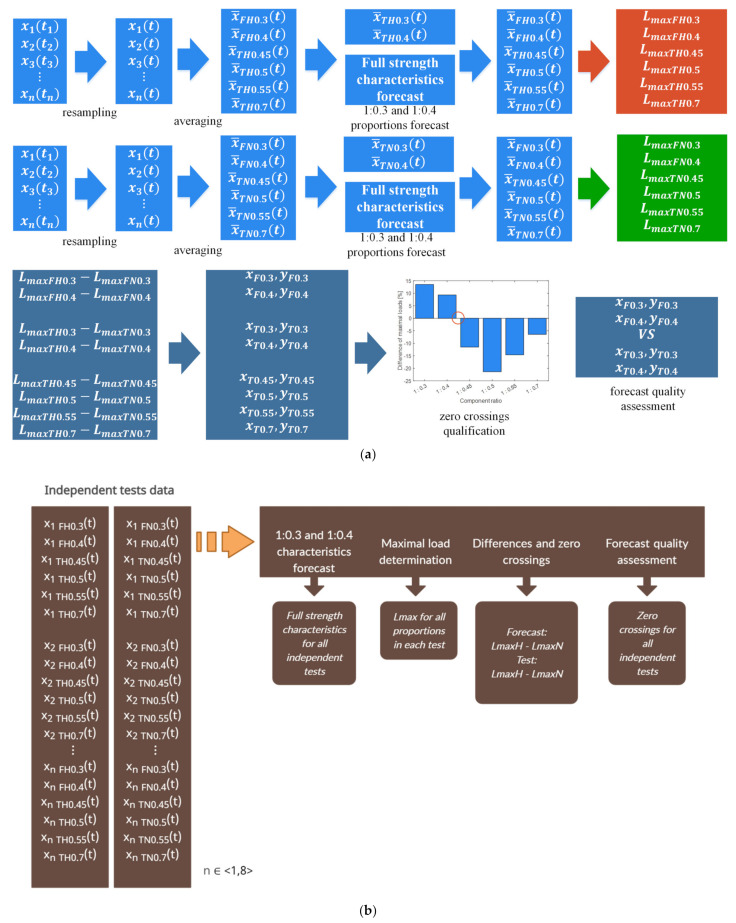
LSTM processing diagram: (**a**) averaged tests and (**b**) independent tests.

The values of the differences present within the assumed mix ratios were used to determine the node points. The zero crossings of the static strength change vs. tested adhesive mix ratio were determined by node interpolation. The diagram of this procedure is shown in Figure 7a.

However, assuming it is feasible to reduce the number of experimental/laboratory tests, complementing the data with forecast values might lead to certain inaccuracies in the specification of adhesive component mix ratios. To more accurately verify the produced results due to the natural measurement discrepancies, it was decided to run a second analysis by testing random samples within the range of the tested mix ratios.

This second analysis included separate forecasting of the strength characteristics for a set of adhesive joints within the tested mix ratios. Like in the previous analysis of the experimental test results, the specimens were sequenced from the highest to the lowest hardener ratio. This time, the share of teaching data in the signal length was between 54% and 76%. The difference was a result of the different lengths of the strength characteristics. No resampling algorithm was implemented in order to avoid distortion in the individual signals. Like in the first analysis, two strength characteristics were forecast for the adhesive joints with the lowest hardener ratios. As in the first analysis, the DLN LSTM integrated in Matlab 2021a was used for the forecasting process.

The first dataset analysed was the specimens with and without the heat-held curing at the test temperature of 70 °C. This dataset was resampled, followed by averaging over the limits of the test temperature, mix ratios, and curing specifications. The next step was to forecast the locations of nodes for the function of strength change following heat holding of the adhesive joints. The zero crossings were determined by interpolation with a cubic spline and a function obtained by the least-squares method. 

The second dataset was random samples in the following range: test temperature, mix ratios, and curing specifications. Similar to the first analysis, the input (teaching) data for the forecast was the trends with the decreasing mix ratios: 1:0.7; 1:0.55; 1:0.5; and 1:0.45. The output was the forecasts of subsequent trends for mix ratios 1:0.4 and 1:0.3. Each resulting trend had the maximum breaking force of the specimen determined separately for the forecasts and the experimental tests. The produced breaking force values for all tested mix ratios were adopted as the nodes for the subsequently interpolated functions. In line with the objective of this paper, each set of specimens had zero crossings determined, denoting the proportions of the components at which holding at heat was no longer relevant to the adhesive joint strength. The processing diagram of the algorithm for independent tests is shown in Figure 7b.

## 3. Results and Discussion

### 3.1. Mechanical Testing

**Figure 8:**
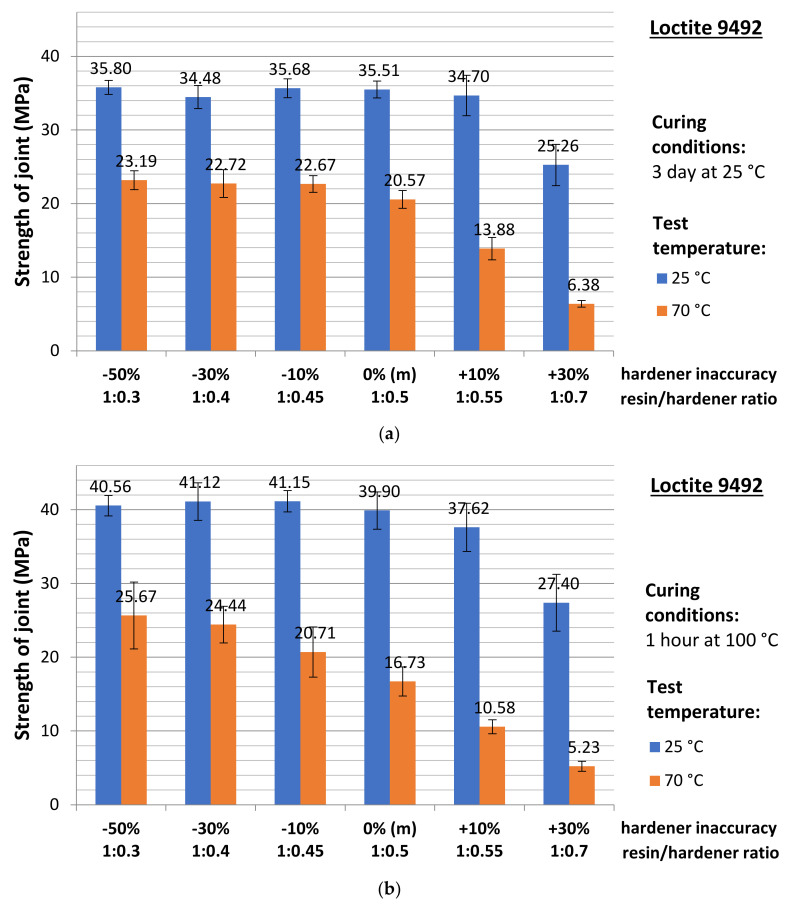
Change in the adhesive joint strength from the test temperature for the joints (**a**) cured without heat holding and (**b**) cured with heat holding.

**Figure 9:**
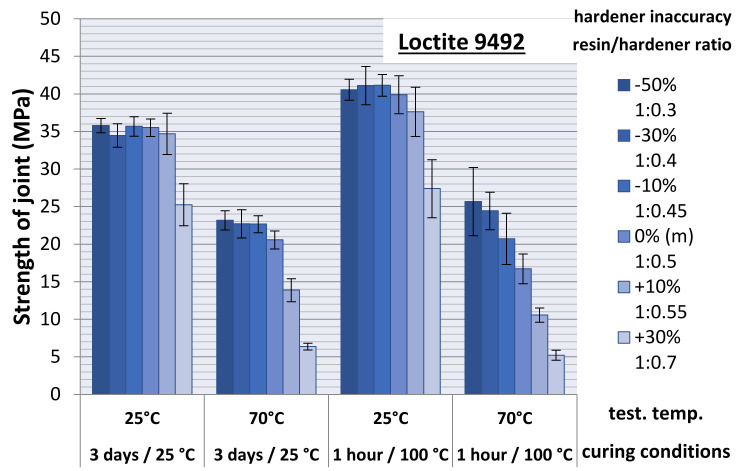
Summary of the strength test results.

The obtained values of the adhesive joint breaking (failure) force were converted to the adhesive joint strength by dividing the force value by the joint (specimen) cross-sectional area. The strength results for the adhesive joints cured at ambient temperature are illustrated in Figure 8a. For the adhesive joints cured by heat holding (for 1 h at 100 °C), the strength results are shown in Figure 8b. Both charts include a slight scatter of the results, being a standard deviation. A summary of all obtained experimental results, allowing for a general comparison of the series between each other, is shown in Figure 9.

For each of the tested adhesive joints, its failure mode was recorded according to ISO 10365 Standard—Designation of main failure patterns [59], with a total of four failure mode types: cohesion failure (within the adhesive joint), adhesion failure (between the adhesive layer and the bonded material), mixed adhesion/cohesion failure, and adhesion/cohesion failure with peel (Table 2).

A summary combining the individual joint series with the resulting tensile strength and failure mode of the joint is shown for the series tested at room temperature in Table 3. Samples tested at elevated temperature are summarised in Table 4.

Preliminary analyses indicate a clearly positive character of the adhesive heat curing process for samples tested at ambient temperature. In each case of the resin/hardener ratio tested, the average compressive strength values obtained are ~8–20% higher for the heat-cured samples. The same is true for samples tested at elevated temperature but only for samples with significant non-hardener addition (−50% and −30%). From a deficiency of about −10% hardener, through the manufacturer’s recommended proportion, to adhesives with excess of hardener, the average strength of the heated joints is lower than that of the unheated ones.

It can also be seen that the nature of the joint failure changes with the amount of hardener in the adhesive formulation. At ambient temperature, an excess of hardener will result in a change in the type of failure from cohesive to cohesive–adhesive. Depending on how the adhesive is cured, this happens differently: for an unheated adhesive, this is at about 30% excess hardener and a heat-cured adhesive shows this change with as little as 10% excess hardener. Interestingly, the correct amount of hardener and its deficiency always resulted in cohesive failure of the joint. The same adhesive compositions tested at elevated temperatures showed a similar trend, with purely cohesive failure being recorded only at very high hardener deficiency (−50%), regardless of whether the bond was heated or not. Smaller values of hardener deficiency, the manufacturers ratio, and a ratio up to 10% hardener excess resulted in joints with mixed failure characteristics. The most significant change in the mode of joint failure between the non-heated and heated adhesive was observed for samples tested at elevated temperature. This can be seen from a hardener deficiency of −10%, where the failure mode changes from 3/4 purely cohesive to mixed (adhesive–cohesive) as a result of samples’ heating. Furthermore, at the manufacturer’s recommended ratio, the mode changes from cohesive-mixed (~50–50%) to approximately ¾ mixed, and at 10% hardener excess from purely mixed to mixed-adhesive (~50–50%). 

A large hardener excess (+30%) resulted in adhesive failure, from adhesive–cohesive with peel for samples that were not heated to purely adhesive failure for samples that were heated while curing. Improved adhesive parameters after heat curing can be explained by the higher mobility of the heated particles of resin, which further supports polymerisation. The adhesive subjected to thermal curing achieves a significantly higher degree of crosslinking, becomes stiffer, and, at the same time, its strength increases. However, for an adhesive tested at elevated temperatures, these changes lead to a deterioration of the adhesive properties and, consequently, to a change in the mode of destruction towards the adhesive one.

**Figure 10:**
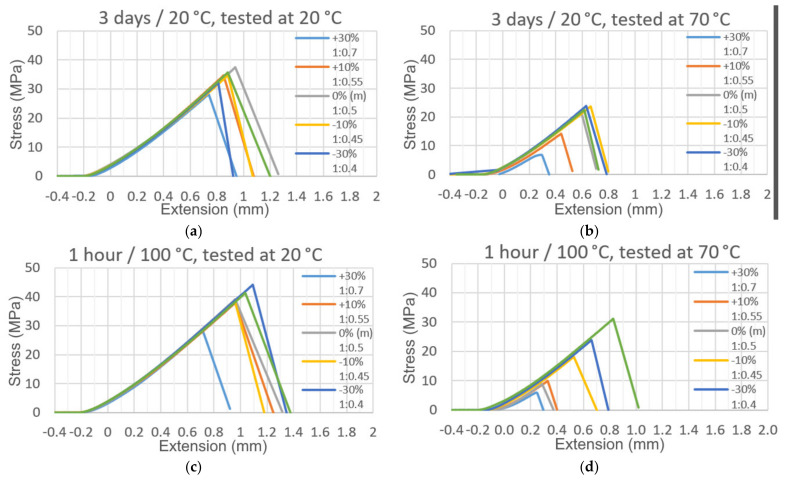
Stress–extension graphs of tested adhesive joints prepared under inaccurate addition of hardener component in adhesive (random samples): (**a**) cured 3 days at 20 °C, tested at 20 °C; (**b**) cured 1 h at 100 °C, tested at 20 °C; (**c**) cured 3 days at 20 °C, tested at 70 °C; and (**d**) cured 1 h at 100 °C, tested at 70 °C.

Figure 10 shows examples of strain-to-stress diagrams obtained during tests at ambient and elevated temperatures of samples made with adhesives of the tested composition and curing conditions.

**Figure 11:**
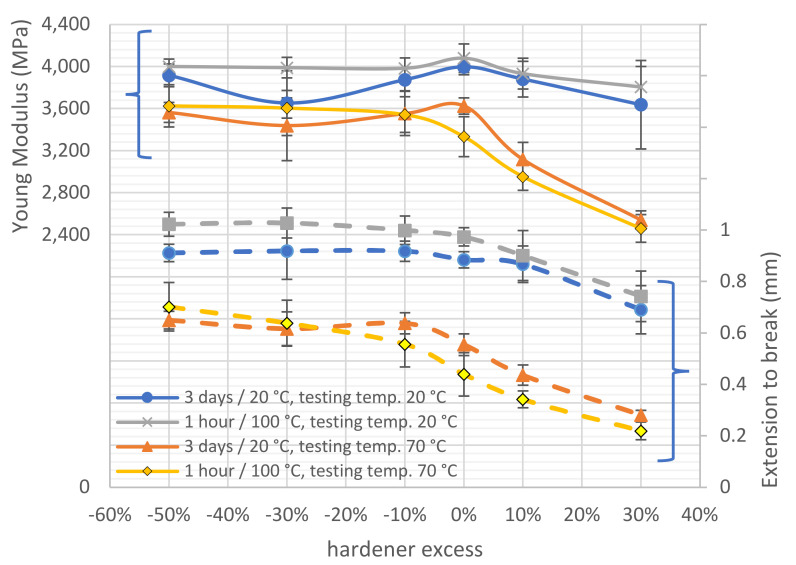
Mean Young’s modulus and mean extension-to-break of tested adhesive joints prepared under inaccurate addition of hardener component in adhesive.

A summary of other important test results, i.e., the value of the maximum strain at which the joint failed or the value of Young’s modulus for joints made with adhesives of different compositions, curing conditions, and test temperatures is shown in Figure 11.

There is a clear decrease in the mean Young’s modulus with an excess of hardener in the adhesive formulation, especially for samples tested at elevated temperatures, with a simultaneous reduction in mean strain to joint failure. A noticeable effect of thermal curing was also observed, which was almost always associated with an increase in the Young’s modulus of the adhesive regardless of its test temperature and the ratio in which the adhesive was made. This can be explained by the higher mobility of the heated particles during curing, aiding polymerisation. An adhesive subjected to thermal curing achieves a significantly higher degree of crosslinking, becomes stiffer, and, at the same time, its cohesive strength increases [61].

**Figure 12:**
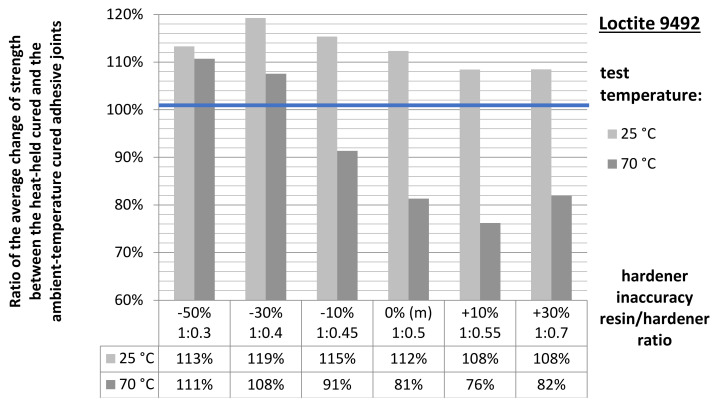
Average strength ratio of the heat cured to the ambient temperature-cured adhesive joints.

The quotient of the produced heat-held cured adhesive joint strength and the ambient temperature-held adhesive joint strength was the average coefficient of change in the adhesive joint strength following heat holding for the adhesive compositions varying in the mix ratio and the test temperature of the joint (Figure 12).

The foregoing summary is only a comparison of the average joint strength values. To understand the relationships between the applied heat holding of cured adhesive joints and their final strength at both various degrees of distortion of the relevant adhesive composition mix ratio and different adhesive joint test temperatures, the produced results underwent mathematical and statistical analysis.

**Figure 13:**
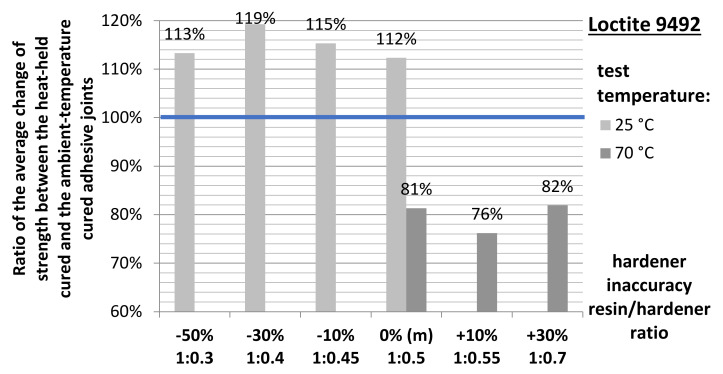
Statistically significant change in the ratio of the average strength between the heat-cured and the ambient temperature-cured adhesive joints.

To verify that significant differences were present between them, checks were done with Student’s t-tests, preceded by the Leven and Brown–Forsythe tests of the equality of variances and a normality test. A graphical comparison from which the comparisons were removed for the series that had no statistically significant difference demonstrated is shown in Figure 13.

In the constant cure cycle for all tests, the rate of polymerisation or cure is dependent on the stoichiometric ratio, r, between the two reacting co-monomers. For adhesives based on diglycidyl ether of bisphenol A (DGEBA) resins cured with 4,4′-diaminodiphenyl sulfone (DDS) hardener, the epoxy-rich ratio resulted in the lowest glass transition temperature (Tg). On the other end, the excess of amine hardener led to lower Tg due to the formation of rings consisting of partially reacted diamine molecules and epoxy chains which increase the free volume of the system [62,63]. Other research [64] attributed the differences in Tg with stoichiometry to be as a direct result of differences in molecular weight between crosslinks, whereby higher Tg resins have a smaller distance between crosslinks. For higher-functionality epoxy resins (tetraglycidyl-4,4’-diaminodiphenylmethane—TGDDM and an anhydride curing agent), the distance between crosslinks was measured and it was lower for resins cured within this stoichiometric range. Additionally, better mechanical properties were obtained for mixtures slightly in excess of epoxy, assuming the improvements were due to an increase in etherification reactions between epoxy groups and hydroxyls, which resulted in an increase in crosslink density [65].

### 3.2. LSTM Forecast Adhesive Joint Strength Values at Elevated Temperature

**Figure 14:**
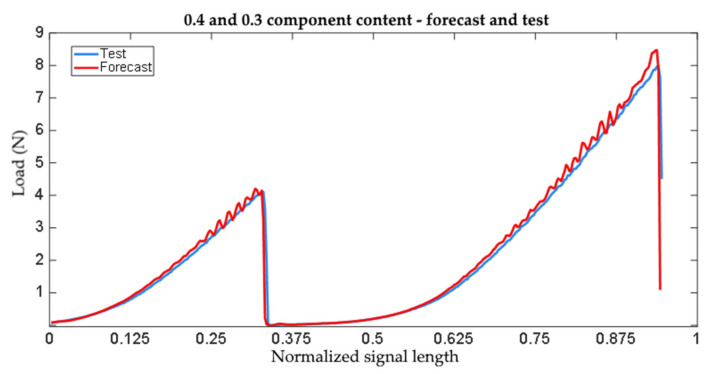
Comparison of the forecast quality to the experimental test values.

The analyses produced forecasts of the full-strength characteristics of the adhesive joints within the limits of the tested mix ratios. An example summary of the tested and forecast trends is shown in Figure 14. 

**Figure 15:**
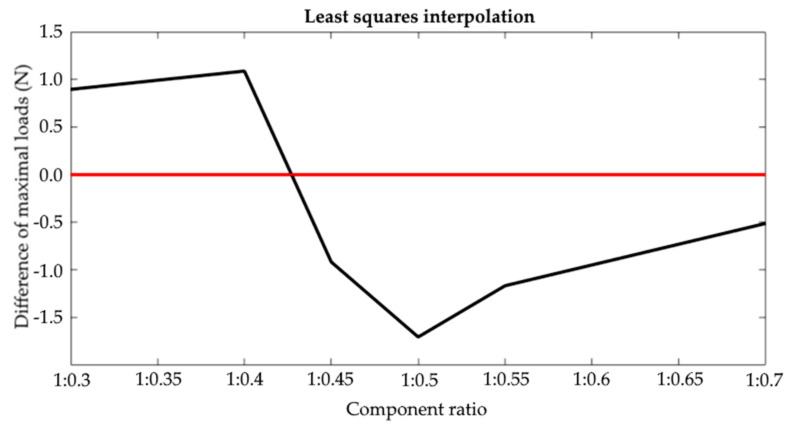
Least-squares interpolation.

**Figure 16:**
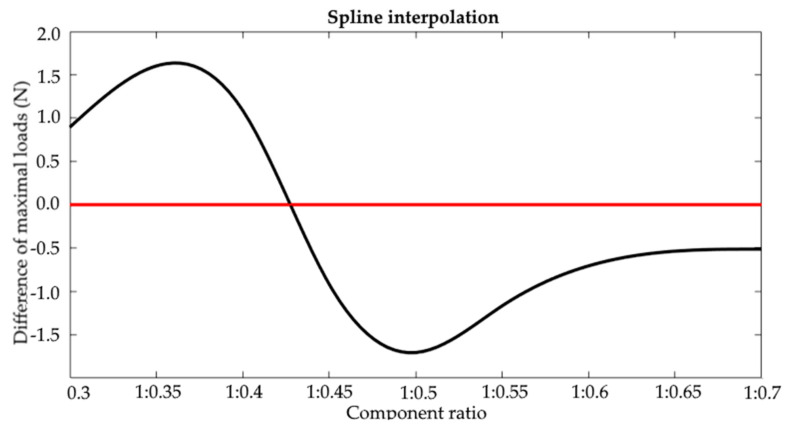
Cubic spline interpolation.

Based on the characteristics produced, differences in the maximum breaking forces were determined between the heat-held cured joints and the ambient temperature-cured joints. The result was the node points with the coordinates which denoted the values of the differences between the maximum breaking forces. The zero crossings were determined by interpolation. This process was applied separately for the values from the experimental tests and the forecasts. Examples of the interpolation results are shown in Figure 15 (least-squares method) and Figure 16 (cubic spline). The full summary of the produced values is shown in Table 5. 

The test results in the second analysis (comprising the averaging of all tests) provided a result consistent with the experimental test (0% error). The tests and simulations proved that 1:0.427 was the mix ratio at which heat curing becomes irrelevant to increasing the tensile strength of adhesive joints.

## 4. Conclusions

This paper demonstrates the feasibility of a numerical method for determining accurate adhesive component mix ratios above which heat holding to cure an adhesive joint will no longer be a significant and positive contributor to the strength of the adhesive joint tested at an elevated temperature. The applied numerical methods, i.e., the LSTM (long short-term memory) artificial recurrent neural network complemented the experimental test methods, allowing for the production of precise results from partial input data without any need for additional and detailed experimental testing. Otherwise, the tests would require more tensile loading of adhesive joint specimens up to their failure. The application of the presented LSTM RNN test methodology will lead to research which is less time and cost intensive.

On the basis of the conducted error analysis in the context of the averaged data and independent tests, and in the context of the minimum number of necessary experiments, it can be concluded that the application of the above method allows for reducing the samples subjected to testing. Taking into account the possibility of the future use of higher sampling rates of signals and thus obtaining longer waveforms (reduction of the amount of necessary learning data with minimal losses in the registration of dynamic link breakage), it is achievable to reduce the amount of input data to 50% of the conducted tests while maintaining the error level within 10%.

The fact that satisfactory analytical results were repeatedly produced leads to the conclusion that the forecasting capabilities are broad and can substitute for other tests which require a considerably higher expense of resources (time and money).

The use of additional methods of mathematical and statistical analysis of data obtained from experimental studies (Taguchi and Response Surface Method RSM) may provide additional information and is planned in further studies.

In the presented results, due to the assumed random nature of the component inaccuracies, it was not analysed whether the adhesive under conditions of inaccuracy of resin and hardener proportions achieves full crosslinking and no quantitative and qualitative relations between this parameter and the resulting strength of the bond were considered. Therefore, as an extension study, differential thermal analysis (DTA), i.e., DSC analysis (differential scanning calorimetry; based on ISO 11357 [66,67]) is planned to determine the level of cure achieved and the time needed to achieve both full cure and thermal parameters, such as the glass transition temperature. The results will allow for extending the knowledge on the thermal processes occurring during the curing process, such as making recommendations on the optimum curing conditions for a joint with an inaccurate (known) resin/hardener component ratio.

## Figures and Tables

**Table 1:** Experimental test plan.

Adhesive Component A/B Mix Ratio (Resin/Hardener)		Curing Conditions		Test Temperature
1.4:1/1:0.71	**x**	3 days/25 °C	**x**	25 °C
1.8:1/1:0.55				
2.0:1/1:0.50(manufacturer-specified “m”)				
2.2:1/1:0.45		1 h/100 °C		70 °C
2.6:1/1:0.40				
3.0:1/1:0.33				

**Table 2:** Failure patterns: (a) CF—cohesion failure, (b) AF + CF—mixed, (c) ACFP—adhesion and cohesion failure with peel, and (d) AF—adhesion failure.

CF	AF + CF	ACFP	AF
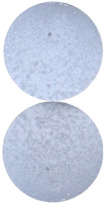	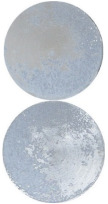	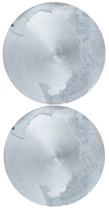	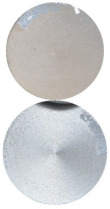

**Table 3:** Failure mode change after heat curing samples tested at 25 °C.

A/B Mix Ratio (Resin/Hardener)	Cured 3 Days at 25 °C		Cured 1 h at 100 °C	
	Mean Tensile Strength (MPa)	Failure Mode	Mean Tensile Strength (MPa)	Failure Mode
−50% 1:0.3	35.80	CF	40.56	CF
−30% 1:0.4	34.48	CF	41.12	CF
−10% 1:0.45	35.68	CF	41.15	CF
m: 1:0.5	35.51	CF	39.90	CF
+10% 1:0.55	34.70	CF	37.62	78% AF + CF, 22% CF
+30% 1:0.7	25.26	89% AF + CF, 11% CF	27.40	AF + CF

**Table 4:** Failure mode change after heat curing samples tested at 70 °C.

A/B Mix Ratio (Resin/Hardener)	Cured 3 Days at 25 °C		Cured 1 h at 100 °C	
	Mean Tensile Strength (MPa)	Failure Mode	Mean Tensile Strength (MPa)	Failure Mode
−50% 1:0.3	23.19	CF	25.67	CF
−30% 1:0.4	22.72	AF + CF	24.44	AF + CF
−10% 1:0.45	22.67	78% CF, 22% AF + CF	20.71	AF + CF
m: 1:0.5	20.57	56% AF + CF, 44% CF	16.73	78% AF + CF, 22% AF
+10% 1:0.55	13.88	AF + CF	10.58	56% AF + CF, 44% AF
+30% 1:0.7	6.38	ACFP	5.23	AF

**Table 5:** Independent test and forecast results.

#	Test	Cubic Spline Forecast	Error (%)	Least-Squares Forecast	Error (%)
1	0.424	0.352	16.98	0.353	16.75
2	0.392	0.400	2.04	0.398	1.53
3	0.465	0.465	0.00	0.465	0.00
4	0.438	0.437	0.23	0.437	0.23
5	0.431	0.432	0.23	0.432	0.23
6	0.417	0.416	0.23	0.417	0.00
7	0.475	0.476	0.23	0.476	0.23
8	0.418	0.417	0.23	0.418	0.00

## Data Availability

Data presented in this study is available from corresponding authors upon request.
